# Comparison of Subjective and Objective Sleep Quality in Patients With Obstructive Sleep Apnea Syndrome

**DOI:** 10.1002/brb3.70759

**Published:** 2025-08-12

**Authors:** Fatemeh Kashaninasab, Mahboobeh Khoozan, Mir Farhad Ghalebandi, Kaveh Alavi

**Affiliations:** ^1^ Mental Health Research Center, Department of Psychiatry, School of Medicine Iran University of Medical Sciences Tehran Iran; ^2^ Mental Health Research Center, Psychosocial Health Research Institute (PHRI), Department of Psychiatry, School of Medicine Iran University of Medical Sciences Tehran Iran

**Keywords:** objective sleep quality, Pittsburgh Sleep Quality Index (PSQI), sleep apnea, sleep efficiency, subjective sleep quality

## Abstract

**Purpose:**

This study aims to compare subjective and objective sleep quality in patients with obstructive sleep apnea (OSA), given the high prevalence of this sleep disorder that can affect sleep quality.

**Method:**

This research enrolled 195 individuals diagnosed with OSA, with an Apnea Hypopnea Index (AHI) of 5 or higher based on polysomnography. Participants completed the Pittsburgh Sleep Quality Index (PSQI) questionnaire for subjective sleep quality. Objective sleep quality derived from sleep efficiency reported in overnight polysomnography.

**Findings:**

An analysis of sleep efficiency showed that 12.8% of people had poor‐quality sleep. The PSQI was also used to measure subjective sleep quality, and 64.1% of respondents reported having poor sleep quality. No significant correlation was observed between sleep efficiency and PSQI scores. Obesity has a negative correlation (*ρ* = −0.168, *p* = 0.019) with sleep efficiency, highlighting the effect of BMI on sleep fragmentation. Male sex was linked to a lower risk of poor objective sleep quality, according to logistic regression analysis (adjusted OR = 0.314, 95% CI = 0.113–0.872). Frequent use of sleeping pills was linked to a lower probability of experiencing subjectively poor sleep quality (adjusted OR = 0.077, 95% CI = 0.024–0.243).

**Conclusion:**

This study highlights that a significant portion of OSA patients have poor sleep quality, subjectively. Although sleep efficiency is an important objective metrics, its lack of correlation with subjective sleep quality in this population highlights the complexity of assessing sleep health and the need for comprehensive evaluation tools in these patients.

## Graphical abstract

This study compares subjective (Pittsburgh Sleep Quality Index) and objective (polysomnography‐derived sleep efficiency) sleep quality in 195 OSA patients. Results reveal significant discordance between the two measures, with 64.1% reporting poor subjective sleep quality while only 12.8% exhibited poor objective sleep efficiency, highlighting the need for comprehensive sleep health evaluations in OSA patients.

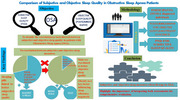

## 1 Introduction

Sleep and breathing are both necessary for sustaining life. We sleep for approximately one‐third of our lives, or 8 h, on average. Our bodies undergo changes while we sleep, which may make us more susceptible to specific sleep disorders (Altena et al. 2022; Xu et al. 2022; Freeman et al. 2020). For example, while our breathing may be normal when we are awake, it is possible to have disruptions in our sleep due to changes that take place when we are asleep (Vgontzas 2008, Spira et al. 2009). The respiratory condition obstructive sleep apnea (OSA) syndrome is one condition that can happen while you are sleeping. OSA is a common sleep disorder in general population, as its prevalence is about 23% in women and 50% in men (Heinzer et al. 2015). In Iran, a nation‐wide epidemiological study using Holland Sleep Disorder Questionnaire showed a prevalence of 38.4% for OSA (Khazaie et al. 2024). Considering STOP‐BANG score of 3 or higher, Foroughi et al. estimated this prevalence as 51.4% in men and 26.5% in women (Foroughi et al. 2017). A systematic review and meta‐analysis by Sarokhani et al. showed a higher prevalence of OSA in some high‐risk population in Iran, as exampled by a prevalence of 74% in patients with any sleep disorder, 61% in diabetic population, and 55% in cardiovascular patients, with overall prevalence of 44% (Sarokhani et al. 2019). However, Sadeghniiat‐Haghighi et al. reported a lower prevalence of 29% (Sadeghniiat‐Haghighi et al. 2023).

One of the main symptoms in patients with OSA is excessive daytime sleepiness. Driving‐related fatal accidents have been linked to OSA and the ensuing excessive fatigue it causes (Lv et al. 2023). OSA is characterized by full or partial airway obstruction brought on by the pharyngeal muscles relaxing while you sleep. This can cause loud snoring or a feeling of choking, as well as frequent awakenings, fragmented sleep, and extreme daytime sleepiness (Mohammadi and M 2012). Clinical signs unique to this syndrome include excessive daytime sleepiness, insomnia, and signs of upper airway obstruction during sleep (snoring, gasping, choking, and breathing pauses). Issues with sleep have a detrimental effect on life quality (Mohammadi and M 2012). OSA disrupts sleep continuity through repeated apneas and subsequent arousals. This fragmentation contributes to impaired sleep quality and excessive daytime sleepiness (Kania et al. 2022).

Overnight polysomnography (PSG) is the standard diagnostic tool for OSA, utilizing various EEG, EOG, EMG, oximetry, and respiratory activities to assess its severity (Tsara et al. 2009, Akashiba et al. 2022). The Epworth Sleepiness Scale questionnaire is a tool used to assess excessive daytime sleepiness. Although this tool is subjective, it is very cost‐effective, convenient, and accessible (Pilcher et al. 2003). Pittsburgh Sleep Quality Index (PSQI (is a common tool used to assess sleep quality, but its correlation with PSG components may be weak, necessitating the development of a more cost‐effective and valid tool for evaluating sleep quality in patients (DiNapoli et al. 2017, Lusic Kalcina et al. 2017).

There was no discernible relationship between self‐reported sleep quality and objective sleep efficiency (SE) in DiNapoli et al.’s (2017) study involving 59 patients with mild cognitive impairment (MCI). Subjective and objective measures of sleep were found to differ in 61% of participants; this discrepancy was found to be influenced by factors such as memory impairment and diagnosis of insomnia (DiNapoli et al. 2017). A 2018 study by Ren et al. found that individuals with OSA and objectively short sleep duration have an 80% increased risk of hypertension, while those with subjectively short sleep duration have a 45% increased risk (Ren et al. 2018).

Johnson et al.’s (2020) study found that sleep disorders like restless legs syndrome, sleep apnea, insomnia, and sleep fragmentation negatively impact sleep quality (Johnson et al. 2020). Duarte et al.’s study found significant differences in sleep perception in adults with OSA (Duarte et al. 2020). A 2021 study by Ma et al. found subjective‐objective sleep discrepancy in patients with OSA and insomnia, with overestimation of delayed sleep onset being the strongest correlation (Ma et al. 2021).

Since numerous reports in articles indicate a high prevalence of sleep problems and daytime sleepiness, but sparse quantitative establishment of comparing subjective and objective sleep parameters in OSA patients, the aim of the present study was to determine the correlation between sleep quality subjectively and objectively in a group of OSA patients. Additionally, unlike previous studies—which have generally reported low correlations between subjective and objective sleep measures in more heterogeneous populations (e.g., study by Ma et al.)—our study specifically examines this discrepancy within patients diagnosed with OSA. By focusing exclusively on this patient group, we aim not only to corroborate the findings of earlier research but also to provide novel insights into the sleep assessment challenges unique to individuals with OSA.

## 2 Methods

This study was a retrospective review of medical files of all individuals referred to the Sleep Laboratory of Rasoul Akram Hospital, for any reason, in a 2‐year period (2020 and 2021). All patients who had been diagnosed with OSA based on apnea hypopnea index (AHI) equal to or greater than 5 in a single‐night in‐lab overnight PSG (Akashiba et al. 2022) enrolled in the study. All PSG tests were interpreted by a sleep medicine fellowship. Medical files with unestablished diagnosis, concurrent primary sleep disorders (including primary insomnia, narcolepsy, REM sleep behavior disorder, etc.), or incomplete files regarding demographic variables or unfulfilled sleep questionnaire(s) were excluded. Ethical approval was obtained from the Research Ethics Committees of Iran University of Medical Sciences (IR.IUMS.REC.1402.241).

In this center, the first step is introducing the laboratory as an academic sleep laboratory that uses and analyzes demographic and clinical data of the clients anonymously in future studies. After providing these explanations, all clients were asked to sign a written consent and then a trained senior psychiatric resident conducted interviews with clients to gather any demographic and clinical (present and past psychiatric and medical history) information. Then a series of relevant questionnaires were presented to the clients to be fulfilled. Clients were free to refuse fulfillment of some questionnaire, if there was no clinical indication for that questionnaire.

We utilized the SOMNOmedics PSG system (SOMNO screen plus), which employs six EEG derivations (F4‐M1, C4‐M1, O2‐M1, F3‐M2, C3‐M2, and O2‐M2), two EOG derivations (E1‐M2 and E2‐M2), and two EMG derivation for the assessment of sleep stages. Additionally, it incorporates oronasal thermal sensor, nasal pressure flow sensor, thoracic and abdominal RIP belt, and pulse oximetry monitoring to evaluate respiratory function during sleep.

AHI is calculated by dividing the total sleep time by the sum of apnea events (complete cessation of breathing for at least 10 s) and hypopnea events (at least 30% reduction in airflow for at least 10 s accompanied by oxygen desaturation of at least 3% or the event is associated with an arousal). AHI is used to classify the severity of OSA during sleep. An index below 5 is considered normal, between 5 and 15 indicates mild apnea, between 15 and 30 indicates moderate apnea, and above 30 is considered severe apnea (Sateia 2014, Sadeghniiat et al. 2011). The inclusion criteria for this study were ability to read and write, age equal to or older than 18 years, and an AHI equal to or greater than 5 on PSG. The exclusion criterion was noncooperation of participants in completing the questionnaires.

To assess subjective sleep quality, all individuals were asked to complete the PSQI (Buysse et al. 1989). The Persian version of the PSQI was used many times in different population in Iran and showed a good reliability (e.g., Cronbach's alpha of 0.77 in Farrahi Moghaddam et al. 2011 and 0.82 in Khosravifar et al. 2015) and had statistically significant correlation with other psychiatric health questionnaire, such as Generalized Health Questionnaire (GHQ‐12), Beck Depression Inventory (BDI‐II), and Epworth Sleep Scale (ESS).

Objective sleep quality is calculated through SE on PSG. SE is determined by the total sleep time divided by the total time spent in bed, multiplied by 100, and a value of 85% or higher is considered normal (DiNapoli et al. 2017). Demographic information including age, gender, marital status, education level, and occupation of patients was extracted from medical files.

Data analysis was carried out using SPSS version 22. Descriptive findings were expressed by standard statistics (mean, frequency, and percentages, as indicated). As data distribution violated normal distribution based on one sample Kolmogorov–Smirnov test, we used Spearman's correlation coefficient to find nonparametric association between quantitative variables. When indicated, data categorization and creating dummy variables were done based on median half in case of individual's age, combining low‐frequency groups in case of marital status and employment, or standard recommendation of the Center for Disease Control and Prevention (CDC) in case of body mass index (BMI) (Centers for Disease Control and Prevention 2024). Multinomial logistic regression analysis—main effects using these dummy variables were used to test predictability of low SE (SE < 85%), based on demographic variables and subjective sleep quality. *p*‐value less than 0.05 was considered statistically significant.

BMI was calculated by dividing an individual's weight in kilograms by the square of their height in meters. For adults aged 20 years or older, BMI categories were classified as follows: underweight (BMI < 18.5); normal weight (18.5–24.9); overweight (25.0–29.9); and obesity class I (30.0–34.9), class II (35.0–39.9), and class III (≥ 40.0), in accordance with World Health Organization (WHO) criteria (Centers for Disease Control and Prevention 2024).

## 3 Results

Our retrospective analysis initially evaluated 213 consecutive patients who underwent PSG at our sleep laboratory. Following comprehensive chart review, we excluded 11 patients (5.2%) with primary sleep diagnoses other than OSA (e.g., narcolepsy). An additional seven patients (3.3%) were excluded due to incomplete or missing questionnaire data that precluded reliable PSQI calculation. The final analytical cohort comprised 195 patients (91.5% of initially screened individuals).

The demographic characteristics of these individuals are shown in Table 1. Among participants, based on AHI, 43 individuals (22.1%) had mild apnea, 50 individuals (25.6%) had moderate apnea, and 102 individuals (52.3%) had severe apnea. Additionally, According to SE, 25 individuals (12.8%) had poor sleep quality, and 169 individuals (86.7%) had desirable sleep quality. SE data are quantitative. PSQI data are subjective. Considering the cutoff score of 5 in PSQI, 125 individuals (64.1%) had poor, and 70 individuals (35.9%) had good sleep quality.

**TABLE 1:** Demographic characteristics of the 195 participants in the study.

Variable	Frequency	Percentage
Gender		
Male	119	61.0
Female	76	39.0
Age group		
Up to 45 years old	106	54.4
Over 45 years old	89	45.6
Marital status		
Single	31	15.9
Married	140	71.8
Divorced	15	7.7
Widow	9	4.6
Occupation		
Freelancer	82	42.1
Employee	33	16.6
Retired	16	8.2
Housewife	54	27.7
Unemployed	10	5.1
Education		
Below diploma	53	27.2
Diploma and bachelor's degree	105	53.8
Master's degree and above	37	19.0
Body mass index (BMI)		
Underweight	1	0.5
Normal BMI	21	10.8
Overweight	58	29.7
Obesity grade 1	45	23.1
Obesity grade 2	29	14.9
Obesity grade 3	41	21.0
Regular use of sleeping pills	64	32.8
Positive history of psychiatric disorders	94	48.2
Positive history of other medical illnesses	83	42.6

**FIGURE 1:**
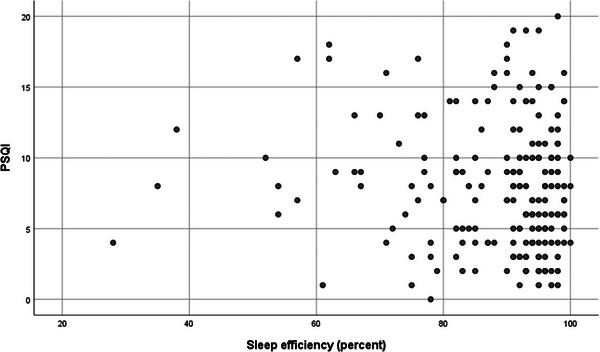
Scatter plot to show correlation between sleep efficiency and Pittsburgh Sleep Quality Index (PSQI). *ρ* = −0.082, *p* = 0.255.

According to Spearman's correlation coefficients (Table 2), the AHI had a weak but significant correlation with age (*ρ* = 0.247, *p* = 0.001). BMI also had a significant correlation with AHI (*ρ* = 0.398, *p* < 0.001). BMI showed a significant negative correlation with SE (*ρ* = −0.168, *p* = 0.019). No significant correlation was observed between SE and PSQI scores (Figure 1).

**TABLE 2:** Spearman's correlation coefficients (Spearman's *ρ*) between age and body mass index (BMI) with sleep quality indices.

	Age	Body mass index (BMI)
Apnea‐Hypopnea Index (AHI)	*ρ* = 0.247, *p* = 0.001	*ρ* = 0.398, *p* < 0.001
Sleep efficiency	*ρ* = 0.046, *p* = 0.520	*ρ* = ‐0.168, *p* = 0.019
Pittsburgh Sleep Quality Index (PSQI)	*ρ* = 0.021, *p* = 0.771	*ρ* = 0.038, *p* = 0.600

In logistic regression analysis (*χ* ^2^ = 17.3, df = 11, *p* = 0.098, Nagelkerke pseudo *R* ^2^ = 0.156), although the overall analysis did not yield a statistically significant model (Table 3), it was shown that being male is associated with a lower likelihood of experiencing low objective sleep quality (adjusted OR = 0.314, 95% CI = 0.113–0.872).

**TABLE 3:** Logistic regression findings for predicting low sleep quality in polysomnography based on sleep efficiency using demographic and clinical variables.

Variable	*B* ± SE	Wald	*p* value	Exp (*B*) (95% CI)
Gender				
Male	(−1.159) ± 0.522	4.936	0.026	0.314 (0.113–0.872)
Age				
Up to 45 years old	(−0.029) ± 0.501	0.003	0.955	0.972 (0.364–2.593)
Apnea‐Hypopnea Index (AHI)				
Mild apnea	(−0.193) ± 0.755	0.066	0.798	0.824 (0.118–3.618)
Moderate apnea	0.456 ± 0.563	0.657	0.418	1.579 (0.524–4.759)
Regular use of sleeping pills				
Yes	(−0.542) ± 0.578	0.880	0.348	0.582 (0.187–1.804)
Positive history of psychiatric disorders				
Yes	(−0.058) ± 0.525	0.012	0.911	0.943 (0.337–2.638)
Positive history of other medical illnesses				
Yes	0.578 ± 0.495	1.361	0.243	1.782 (0.675–4.705)
Body mass index (BMI)				
Normal weight or underweight	(−1.466) ± 1.125	1.699	0.192	0.231 (0.250–2.093)
Overweight	(−0.539) ± 0.612	0.778	0.378	0.583 (0.176–1.934)
Marital status				
Married	(−1.016) ± 0.593	2.932	0.087	2.762 (0.863–8.835)
Pittsburgh Sleep Quality Index (PSQI)				
Low sleep quality	0.215 ± 0.561	0.147	0.701	1.240 (0.413–3.727)

In the second logistic regression analysis (*χ* ^2^ = 54.5, df = 11, *p* < 0.001, Nagelkerke pseudo *R* ^2^ = 0.335), it was found that regular use of sleeping pills is associated with a lower likelihood of reporting poor subjective sleep quality (adjusted OR = 0.077, 95% CI = 0.024–0.243; Table 4).

**TABLE 4:** Logistic regression findings for predicting low sleep quality based on Pittsburgh Sleep Quality Index (PSQI) using demographic and clinical variables.

Variable	*B* ± SE	Exp (*B*) (CI 95%)	Wald	*p* value
Gender				
Male	0.428 ± 0.409	1.534 (0.689–3.418)	1.098	0.295
Age				
Up to 45 years old	(−0.015) ± 0.381	0.985 (0.467–2.080)	0.001	0.969
Apnea‐Hypopnea Index (AHI)				
Mild apnea	0.554 ± 0.039	1.116 (0.376–3.306)	0.039	0.843
Moderate apnea	0.464 ± 2.147	1.973 (0.795–4.894)	2.147	0.143
Regular use of sleeping pills				
Yes	(−2.563) ± 0.586	0.077 (0.024–0.243)	19.102	< 0.001
Positive history of psychiatric disorders				
Yes	(−0.729) ± 0.375	0.482 (0.232–1.005)	3.787	0.052
Positive history of other medical illnesses				
Yes	(−0.609) ± 0.393	0.544 (0.252–1.176)	2.395	0.122
Body mass index (BMI)				
Normal weight or underweight	(−0.882) ± 0.464	0.414 (0.117–1.462)	1.877	0.171
Overweight	(−0.173) ± 0.426	0.841 (0.365–1.940)	0.165	0.685
Marital Status				
Married	0.112 ± 0.397	1.119 (0.514–2.437)	0.080	0.777
Pittsburgh Sleep Quality Index (PSQI)				
Low sleep quality	0.371 ± 0.569	1.449 (0.475–4.420)	0.425	0.515

## 4 Discussion

Our study revealed a striking dissonance between subjective sleep quality and SE, one of the key objective measures of sleep quality, among patients with OSA. While nearly two‐thirds of participants reported poor sleep quality based on the PSQI, only 12% exhibited poor SE as determined by PSG. This incongruity underscores the profound impact of OSA on perceived sleep quality, contrasting with its comparatively limited influence on objective sleep assessments. The observed discrepancy between subjective and objective sleep quality highlights the complex nature of sleep disorders like OSA. Despite objective evidence of disrupted sleep patterns, individuals with OSA may not subjectively perceive the severity of their sleep disturbances (Frangopoulos et al. 2021, Bianchi et al. 2013). Our findings regarding this discrepancy align with previous research (Ma et al. 2021; DiNapoli et al. 2017). However, our study specifically focused on patients with OSA, whereas Ma et al. examined individuals with comorbid insomnia and OSA and DiNapoli et al. investigated sleep discrepancies in older adults with MCI and subsyndromal depression (DiNapoli et al. 2017, Ma et al. 2021)

Patients with OSA tend to overestimate their total sleep time, likely due to factors such as sleep fragmentation, increased daytime sleepiness, and disruptions caused by respiratory events. These factors may contribute to the subjective–objective mismatch. Additionally, patients with insomnia often underestimate their total sleep time while overestimating the time required to fall asleep, reflecting a more negative perception of their sleep quality. Comorbid insomnia in OSA patients may further exacerbate this mismatch (Ma et al. 2021). This finding emphasizes the importance of considering both subjective and objective measures in evaluating sleep health, as subjective perceptions may not always align with objective physiological indices. Also, in terms of subjective sleep quality assessed by the PSQI, a substantial proportion of participants reported poor sleep quality.

Interestingly, our study found no significant correlation between objective and subjective measures of sleep quality among individuals with OSA. This lack of association suggests that objective metrics, such as SE measured by PSG, may not accurately reflect individuals' subjective experiences of sleep disturbances. This discrepancy between subjective and objective sleep quality in OSA patients has been noted in similar studies, underscoring the complexity of assessing sleep health in this population (Cho et al. 2022, Trimmel et al. 2021). Clinical expectations of high SE in individuals with OSA due to prolonged sleep duration may not align with subjective reports of poor sleep quality. This disparity challenges the notion that SE alone is a sufficient indicator of sleep quality in OSA patients.

Our findings revealed a significant association between SE measured by PSG and BMI among individuals with OSA. This highlights the role of obesity in contributing to sleep fragmentation and disturbances in this population. Moreover, our regression analysis demonstrated that BMI was not independently predictive of objective sleep quality, suggesting that other factors may also influence SE in individuals with OSA. Conversely, male gender emerged as a significant predictor of SE, even after controlling for background and clinical variables. Logistic regression analysis revealed intriguing associations, with male sex being associated with a lower likelihood of experiencing low objective sleep quality. This observation aligns with existing literature suggesting sex‐based differences in sleep apnea prevalence and severity (S. Y. Lee et al. 2020; S. Lee et al. 2021).

Furthermore, there was a noticeably stronger correlation between AHI and BMI, indicating the established link between obesity and sleep apnea. A higher BMI was linked to more severe apnea, highlighting the importance of weight control in reducing sleep‐related respiratory problems. The study discovered a significant inverse relationship between BMI and SE in terms of sleep quality, indicating that a higher BMI is associated with lower SE. This supports the idea that obesity can cause sleep architecture to be disrupted and lead to fragmentation of sleep.

Our regression analysis revealed a noteworthy association between subjectively poor sleep quality and the use of sleeping pills among individuals with OSA. This finding suggests the possibility of comorbid sleep disorders, such as chronic insomnia, in this population, which may exacerbate sleep disturbances and contribute to the perception of poor sleep quality. However, it's important to note that the use of sleeping pills in individuals with OSA may not significantly improve subjective sleep quality and could potentially worsen symptoms, particularly in cases of exacerbation of sleep apnea (Kang et al. 2016, Laranjeira et al. 2018). Furthermore, regular use of sleeping pills was associated with a lower likelihood of reporting poor subjective sleep quality. This finding raises questions about the role of pharmacological interventions in improving perceived sleep quality among individuals with sleep disorders.

Additionally, our study found significant correlations between the AHI and both age and BMI. These findings align with previous research, which demonstrate the age‐related and obesity‐related risks associated with OSA severity (Mayra et al. 2022). However, further investigation comparing our results with similar studies is warranted to better understand the relationship between demographic factors and OSA severity. It is interesting to note that the study found a small but statistically significant positive correlation between AHI and age, meaning that older people were more likely to score higher on the AHI scale. This finding implies the significance of taking age‐related changes in sleep quality into account when evaluating patients with suspected sleep apnea. Age is known to be a risk factor for sleep‐disordered breathing.

Present study underscores the profound impact of OSA syndrome on individuals' sleep quality. The discordance between subjective and objective measures of sleep quality in this population highlights the complexity of assessing sleep health in OSA patients. SE emerges as a valuable metric for evaluating sleep quality in individuals without OSA, but its suitability may be limited in those with the disorder. Demographic factors, such as age and BMI, play a significant role in OSA severity and should be considered in comprehensive sleep evaluations and tailored interventions aimed at improving sleep health in this population.

Based on our findings, it is suggested that relying solely on objective assessments may be insufficient for evaluating sleep quality in patients with OSA. It is recommended that clinicians incorporate subjective evaluation such as detailed sleep quality questionnaires and clinical interviews to gain a comprehensive understanding of these patients’ sleep quality. Additionally, comorbid conditions, such as insomnia, which may influence patients’ self‐reported sleep quality, should be thoroughly evaluated in this population.

### 4.1 Limitations

This study has several limitations that warrant consideration. First, the cross‐sectional design inherently precludes the establishment of causal relationships between variables. While our findings provide valuable insights into associations, longitudinal studies are required to infer causality.

Second, our reliance on single‐night laboratory PSG introduces potential measurement bias. A single night's data may not fully capture a patient's habitual sleep patterns due to the “first‐night effect,” a phenomenon in which unfamiliar laboratory environments alter sleep architecture and it could worsen sleep quality (Byun et al. 2019). Future studies incorporating multiple nights PSG could mitigate this limitation.

Third, the study population was derived from a single hospital‐based sleep laboratory, which may introduce selection bias, which potentially limits the generalizability of our results. To enhance external validity, multicenter studies with diverse demographic and clinical profiles are recommended.

Finally, although additional PSG metrics were available, they were not incorporated into this analysis; thus, we focused only on SE as objective measures and this scope may significantly limit the validity and comprehensiveness of conclusions regarding the mismatch between objective and subjective sleep assessments and should be addressed in future studies.

## 5 Conclusion

The current study provides insight into the point that OSA affects the quality of sleep as based on subjective sleep assessments using PSQI, a sizable portion of participants reported having poor sleep quality. Furthermore, our study found no significant correlation between subjective measures of sleep quality and objective measure of SE among individuals with OSA. This lack of association suggests that objective metrics, such as SE measured by PSG, may not accurately reflect individuals' subjective experiences of sleep disturbances. This discrepancy between subjective and objective sleep quality in OSA patients underscores the complexity of assessing sleep health in this population and emphasized considering other sleep comorbidities, other mental conditions may alter sleep quality satisfaction and patients’ report regarding the results of treatment as an independent outcome variable.

## Author Contributions

**Fatemeh Kashaninasab**: conceptualization, investigation, writing – original draft, methodology, writing – review and editing, validation, project administration, data curation, supervision, resources. **Mahboobeh Khoozan**: conceptualization, investigation, writing – original draft, methodology, validation, writing – review and editing, project administration, formal analysis, data curation, supervision. **Mir Farhad Ghalebandi**: conceptualization, investigation, writing – original draft, validation, visualization, writing – review and editing, project administration, supervision, resources. **Kaveh Alavi**: conceptualization, writing – original draft, methodology, validation, visualization, software, formal analysis, supervision.

## Conflicts of Interest

The authors declare no conflicts of interest.

## Peer Review

The peer review history for this article is available at https://publons.com/publon/10.1002/brb3.70759.

## Data Availability

The data that support the findings of this study are available from the corresponding author upon reasonable request.
